# “Ask Ernö”: a self-learning tool for assignment and prediction of nuclear magnetic resonance spectra

**DOI:** 10.1186/s13321-016-0134-6

**Published:** 2016-05-05

**Authors:** Andrés M. Castillo, Andrés Bernal, Reiner Dieden, Luc Patiny, Julien Wist

**Affiliations:** Facultad de Ingeniería, Universidad Nacional de Colombia, Bogotá D.C., Colombia; Chemistry Department, Universidad del Valle, Cali, Colombia; Analytical Research Center, R&T - Flavors Division EAME, Symrise, Holzminden, Germany; Institute of Chemical Sciences and Engineering, Ecole Polytechnique Fédérale de Lausanne, 1015 Lausanne, Switzerland

**Keywords:** Nuclear magnetic resonance, Automatic assignment, Chemical shift prediction, Peak-picking, Machine learning, HOSE codes

## Abstract

**Background:**

We present “*Ask Ernö*”, a self-learning system for the automatic analysis of NMR spectra, consisting of integrated chemical shift assignment and prediction tools. The output of the automatic assignment component initializes and improves a database of assigned protons that is used by the chemical shift predictor. In turn, the predictions provided by the latter facilitate improvement of the assignment process. Iteration on these steps allows *Ask Ernö* to improve its ability to assign and predict spectra without any prior knowledge or assistance from human experts.

**Results:**

This concept was tested by training such a system with a dataset of 2341 molecules and their ^1^H-NMR spectra, and evaluating the accuracy of chemical shift predictions on a test set of 298 partially assigned molecules (2007 assigned protons). After 10 iterations, *Ask Ernö* was able to decrease its prediction error by 17 %, reaching an average error of 0.265 ppm. Over 60 % of the test chemical shifts were predicted within 0.2 ppm, while only 5 % still presented a prediction error of more than 1 ppm.

**Conclusions:**

*Ask Ernö* introduces an innovative approach to automatic NMR analysis that constantly learns and improves when provided with new data. Furthermore, it completely avoids the need for manually assigned spectra. This system has the potential to be turned into a fully autonomous tool able to compete with the best alternatives currently available.

**Graphical abstract:**
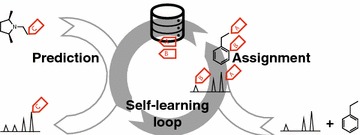
Self-learning loop. Any progress in the prediction (forward problem) will improve the assignment ability (reverse problem) and vice versa.

**Electronic supplementary material:**

The online version of this article (doi:10.1186/s13321-016-0134-6) contains supplementary material, which is available to authorized users.

## Background

The automation of chemical analysis by nuclear magnetic resonance (NMR) spins around two problems: the *forward problem* of predicting the NMR spectra of a given molecule, and the *inverse problem* of elucidating the molecular structure that generates a given experimental spectrum. The forward problem is solved, in principle, by quantum mechanics: molecular structure determines a unique Hamiltonian from which all measurable NMR parameters can be computed. However, this solution is impractical in most cases of interest. First, the ab initio prediction on a personal computer of the NMR parameters (chemical shifts and scalar couplings) for a small molecule takes at least as long as the actual experiments. Second, an isolated molecule is actually a very poor model for a real NMR spin-system in solution, as it ignores solvent effects and the existence of multiple conformations. Accounting for these issues, if possible, would imply even longer calculations. Thus, ab initio prediction of NMR parameters is not a suitable approach for automatic analysis of NMR data.

In practice, the forward problem of NMR prediction is handled by semi-empirical methods based on previous knowledge about typical chemical shifts. Indeed, several commercial packages exist that perform NMR prediction based on models adjusted to large databases of observed chemical shifts [1–9]. To build such databases nuclei must be assigned to observed chemical shifts, a task that concerns the much more challenging inverse problem. Furthermore, predicted chemical shifts play an important role in the assignment process as well. The two problems are thus strongly related, a fact that poses an important limitation to the automation of NMR analysis. This reflects in existing computational tools for NMR elucidation and assignment: either they are not fully-automatic, requiring preliminary analysis by the user [10, 11], or resort to chemical shift predictions [10, 12–18] that rely on databases of spectra assigned ‘manually’ by trained experts. Regardless of the approach, a significant amount of labour is involved that is certainly not devoid of human errors.

We can turn this issue around by noting that the strong relation between the forward and inverse problems means that progress in one direction improves the other [19]. Indeed, successful assignment of a spectrum generates information that can enrich the database used by an NMR predictor. In the opposite direction, more accurate and reliable chemical shift predictions facilitate rejection of non-viable assignments.

This relation then allows to devise a fully automatic assignment and prediction system that progressively improves its capabilities. Learning *ex nihilo*, however, requires an automatic assignment method that is able to assign several spectra without resorting to chemical shift prediction. We developed such method elsewhere [20] and use it here to create *Ask Ernö,*^1^ a fully autonomous assignment—prediction program.

## Methods

**Fig. 1:**
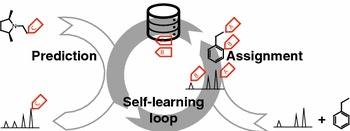
The logic behind *Ask Ernö*. The automatic assignment of nuclei to their signals (*right*) produces entries to a database (*mid*) for chemical shift prediction (*left*). Predicted chemical shifts in turn provide further restrictions for assignment. *Ask Ernö* is trained by repeatedly looping on this assignment-prediction cycle

The concept behind *Ask Ernö* is summarized in Fig. 1. Automatic assignment of a nucleus in a molecule associates a substructure (the nucleus and its surroundings) with an observed chemical shift. This information can be stored in a database and used to predict chemical shifts. As the database grows, the accuracy of the predictor improves. The improved predictor, in turn, provides better chemical shift constraints to be used in subsequent assignments. *Ask Ernö* learns by running repeated assignment cycles on a given training set, using each new assignment to improve its predictions in the next cycle.

*Ask Ernö* was implemented as a proof of concept rather than a full-fleshed assignment and prediction tool. For this reason, it was designed with small molecules in mind and tested only with ^1^H NMR data.

### Chemical shift prediction

**Fig. 2:**
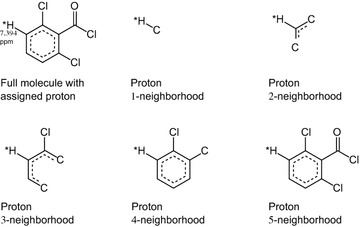
*n*-spheres with radius 1–5 of a proton assigned to a chemical shift of 7.394 ppm. *Dotted curves* indicate aromatic bonds
- *δ*: an observed chemical shift value for the proton.

Each entry in the database for chemical shift prediction consists of two terms:

- *F:* a molecular fragment around a proton, comprising the substructure spanned by all atoms up to *n* bonds from it, with *n* ∊ {1, 2,…}. We refer to this fragment as the *n*-*sphere around the proton* and to *n* as its *radius* or *size* (see Fig. 2). These fragments are stored as Hierarchically Ordered Spherical description of Environment (HOSE) codes [22].

These registers are generated by automatic assignment of experimental spectra (see Learning for details). Since the same fragment *F* may be observed and assigned in different molecules, multiple entries may exist for each fragment.

Predictions were done using the HOSE-based methodology developed in CSEARCH for ^13^C-NMR [24] and later implemented in Modgraph NMRPredict [25]. For ^1^H-NMR it works as follows: the predictor spans the *n*-sphere of radius *n*_max_ around the proton of interest, encodes the resulting fragment as a HOSE code, and queries it on the database. If the query is successful, the median $\underline{\delta }$ over all matches is returned as the predicted chemical shift, with an uncertainty $\varepsilon$ equal to their standard deviation. If no matching entries are found, a new query is sent for the *n*-sphere of radius *n*_max_ − 1 around the proton and so on, until a successful match is found or the radius of the sphere is below *n*_min_. In the latter case the predictor returns a failed status.

### Assignment

We used an automatic assignment method previously described in [20] that performs fully automatic peak-picking and assignment of chemical shifts based on peak integrals (signal intensity), 2D spin–spin correlations, and chemical shifts. The assignment routine uses a symmetry-constrained branch and bound optimization that achieves a thorough exploration of the whole solution space. The result is a list of of assignments, ranked according to how well they fit the observed data. This automatic assigner has been shown to yield good results even if no chemical shift data is provided, which is of great importance for the present development.

Since we only used ^1^H-NMR spectra, assignments were performed exclusively on the basis of integration and chemical shift data. The auto-assigner was configured to seek for assignments that perfectly matched the observed integrals (rounded to the closest integer), and that matched the predicted and observed chemical shifts (when available) with an error no greater than 3 times the prediction’s uncertainty at the current iteration. For this purpose, the uncertainty was estimated as the standard deviation of the sample of observed chemical shifts on which the prediction is based (see *Chemical shift prediction* above), multiplied by the following factor:

$$1 + n^{ - I/2}$$

where *n* is the size of the sample and *I* is the index of the current iteration (see *Learning* below). This factor contributes significantly to controlling the propagation of error, since the standard deviation is a poor estimator of the uncertainty for small *n* or *I*. Furthermore, for predictions based on less than two matches the allowed chemical shift error was set to the maximum of 20 ppm, considering that no reasonable estimation of uncertainty is possible in that situation.

### Learning

The learning algorithm is based on a self-organizing map and consists of a recursive cycle on the training dataset, which is repeated until nothing new is learnt. The first learning iteration starts by running the automatic assignment algorithm without taking chemical shifts into account. We refer to it as *iteration 0*. Redundancy (e.g. multiple occurrences of methyl groups) is expected so that several possible assignments may be found for any given molecule; this is particularly true when no spin–spin correlation data (2D NMR experiments) is available. Though a unique solution is unlikely, it is often possible to find some nuclei—chemical shift dyads that are present in all assignments computed for a molecule and that can thus be assumed to be correct (see Table 1). These dyads are learnt by creating database entries for the corresponding *n*-spheres, with *n* = *n*_min_,…, *n*_max_ (see *Chemical shift prediction* above).

**Table 1:** Results of the automatic assignment of a 5-proton molecule performed based on integrals exclusively

	Proton *a*	Proton *b*	Proton *c*	Proton *d*	Proton *e*
1	1.30	2.52	4.16	7.47	8.27
2	2.52	1.30	4.16	7.47	8.27
3	1.30	2.52	4.16	8.27	7.47
4	2.52	1.30	4.16	8.27	7.47

Despite the ambiguity introduced by the existence of 4 possible solutions, assignment of proton *c* to the peak at 4.16 ppm is present in all of them. This nucleus—chemical shift pair is thus deemed correct and selected to be learnt

Completing this process on all molecules of the training set finishes iteration 0. The system then proceeds with iterations 1, 2, etc., in which newly learnt chemical shifts are used as additional restrictions for subsequent assignments. Database entries are batch-generated, that is, chemical shifts learnt in one cycle are only available starting from the next one. We found in preliminary tests that this approach slows down the learning process but yields better results than the “on-line” approach. Learning continues until two consecutive iterations yield no improvement.

## Experimental

*Ask Ernö* was implemented in Java (automatic assigner), MySQL (prediction database) and JavaScript (chemical shift predictor, self-learning loop and integration of the system’s components). The project is open source and available on GitHub [23], along with links to the data used for training and testing. A web service is available at https://www.cheminfo.org/flavor/askerno/index.html for anyone willing to evaluate the system.

The data used for the evaluation consisted of 2639 molecules along with their experimental ^1^H-NMR spectra. Examples of these spectra are included as Additional file 1. The dataset was assembled by random sampling from the Maybridge catalogue (2198 selected registers) and from our own library (441 selected registers). Data was split in a training set (2341 molecules, Additional file 2) and a test set (298 molecules, Additional file 3). No assignment information was provided along with the training set. Spectra in the test set were manually assigned to determine the reference experimental chemical shift values for the calculation of prediction error. Not all protons in the set were assigned. Most remarkably, labile protons were avoided considering that they are known to pose challenges to the components of *Ask Ernö* [20] and that we intended to evaluate the potential and issues of the self-learning loop rather than those of its components. Overall, 2007 assigned protons were used to test *Ask Ernö*’s predictions.

Ten iterations of training were run, with *n*_*max*_ = 4 and *n*_*min*_ = 2. At the end of each iteration, chemical shifts for the test molecules were predicted and compared with the observed values.

## Results and discussion

**Fig. 3:**
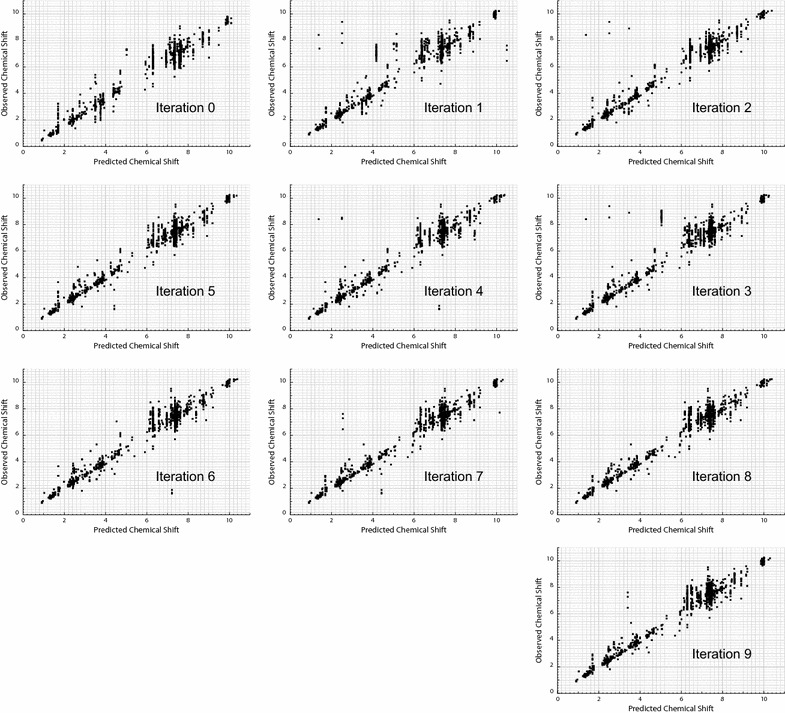
Correlation between observed and predicted chemical shift values for the test molecules at each iteration of the training loop

Figure 3 shows the evolution of the correlation between predicted and observed chemical shifts through 10 iterations. It can be seen that predictions oscillate from one iteration to the other as they converge towards the observed value (diagonal). Indeed, at the last iteration, most predictions accumulate close to the diagonal, though a few large errors persist.

To get a more detailed picture of *Ask Ernö*’s performance and learning process we looked at three indicators: prediction error, prediction uncertainty, and the fraction of chemical shifts from the test set that could be predicted.

### Prediction error

**Fig. 4:**
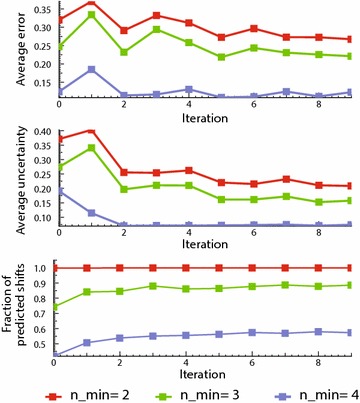
Evolution during the training loop of prediction error (*top*), prediction uncertainty (*middle*) and fraction of predicted chemical shifts (*bottom*) for the test molecules

The overall prediction error is expected to decrease as the system iterates, and final errors to be the lowest possible. Figure 4 (top) shows the evolution of the average error across the iterations for *n*_*min*_ = 2, 3, 4. It is found that larger *n*_*min*_ values yield lower errors, but also that it improves less through each iteration (slower learning). Indeed, a larger sphere radius gives a better representation of the magnetic environment of the proton of interest, producing a more accurate prediction that can hardly be improved. For smaller fragments the distribution of observed chemical shifts is wider, so there is more room for improvement. Thus, as the system iterates and the database of fragments grows, the average chemical shift of matching fragments moves closer to the true mean of the full distribution, lowering the average prediction error. For *n*_*min*_ = 2 this error decreased by 17 % across 10 cycles, for a final value of 0.265 ppm.

**Fig. 5:**
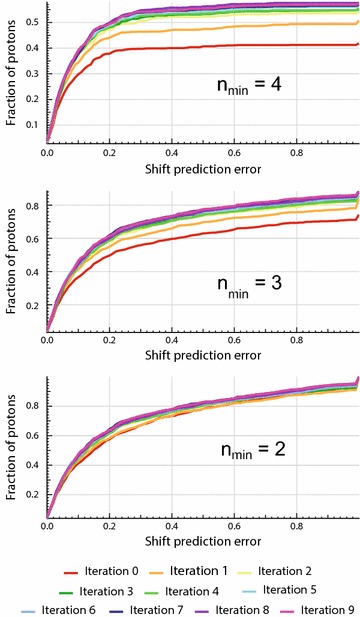
Evolution of the cumulative error distributions during training. The fraction of predictions is given relative to the total number of protons in the test set for which chemical shift can be predicted (2007 protons in total). To generate these curves, the set of chemical shift prediction errors was split into 100 bins of 0.01 ppm, plus a last bin containing predictions with an error equal or greater than 1 ppm. This last bin being larger explains the sudden increase observed at the end of the curves

Since the average error can be dominated by a few predictions with large errors, the cumulative error distributions were plotted (Fig. 5). It can be seen that larger *n*_*min*_ values yield a higher number of accurate predictions (<0.2 ppm) and fewer predictions with high error (>1 ppm). Also, the number of accurate predictions grows faster with larger *n*_*min*_ (12 % at *n*_*min*_ = 4 vs. 4 % at *n*_*min*_ = 2), while the number of less accurate predictions reduces more slowly (<1 % for *n*_*min*_ = 4 vs. 4 % at *n*_*min*_ = 2). This is consistent with the observed behavior of the average error and again is explained by the naturally higher accuracy of predictions achieved with larger *n*-spheres. In the end, with *n*_*min*_ = 2, over 60 % of the tested chemical shifts were predicted with less than 0.2 ppm error, and only 5 % of them were found with error exceeding 1 ppm.

### Prediction uncertainty

In *Ask Ernö*, the uncertainty of a prediction is associated with the standard error of the distribution of chemical shifts of matching fragments (see Methods, Chemical shift prediction). While the prediction error validates the results against an external reference (the correct chemical shifts), the uncertainty provides an internal validation. It is expected that as the system learns it gives predictions with lower uncertainty.

Figure 4 (mid) presents the evolution of this statistic through the training. It can be seen that the uncertainty quickly decreases, reaching a limit value. Both the rate and limit value are related to *n*_*min*_: the smaller *n*_*min*_ the faster the uncertainty decreases and the lower it reaches (0.23 ppm for *n*_*min*_ = 2 and < 0.1 ppm for *n*_*min*_ = 4).

Note that this limit is nothing but the standard deviation of the distribution of chemical shifts on the population of all possible *n*-spheres for the corresponding *n*_*min*_. This allows for an interesting interpretation of the limit uncertainty as the theoretical best that *Ask Ernö* can achieve. Noting how the final average error in Fig. 4 (top) is above the limit uncertainty in Fig. 4 (mid), we conjecture that *Ask Ernö’*’s accuracy can still be improved by around 13 % through further training with more data.

### Amount of predicted chemical shifts

For a chemical shift to be predicted, it is necessary that a matching substructure is found in the database. The fraction of chemical shifts from the test set that can be predicted then constitutes a third descriptor of learning. Figure 4 (bottom) shows that though larger *n*-spheres provide better predictions, they only cover around half of the test problems (54 % for *n*_*min*_ = 4 at the end of learning). Including predictions with *n*_*min*_ = 3 and *n*_*min*_ = 2 allows for a major leap in coverage, up to 85 and 99 %. It is clear that no significant improvement can be gained by considering 1-spheres.

It is worth noting that the fraction of predictions with larger *n*-spheres increases by 13 % during training. This is pivotal to *Ask Ernö*’s performance: as its database grows, larger *n*-sphere matches becomes possible, which translates into a higher number of more accurate predictions.

### Sources of error

**Fig. 6:**
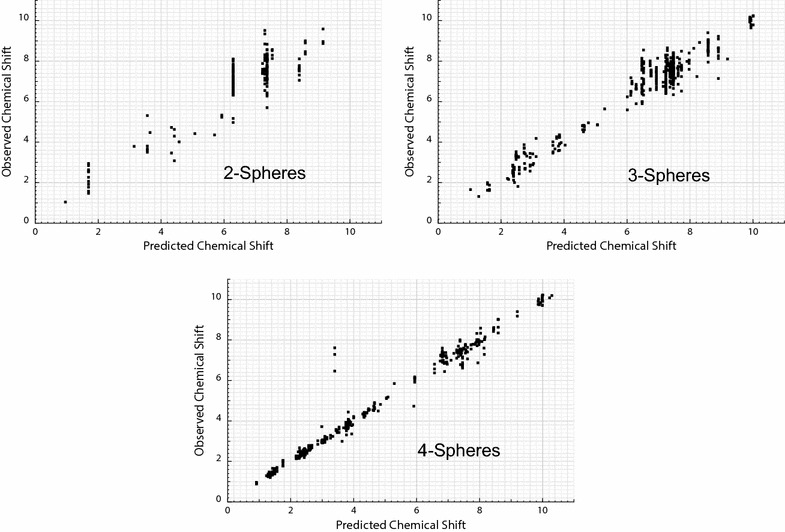
Correlation between observed and predicted chemical shift values after learning for different sphere radius (iteration 9)

*Ask Ernö* is particularly prone to errors when working with structures underrepresented in the training set. For instance, consider a prediction based on a small fragment that is present in numerous molecules of the training set. Since this small fragment is unable to properly account for all relevant interactions, it is associated with a broad range of chemical shifts and the uncertainty of the prediction is very high. Although such fragments are only used until a bigger match is found, no better match will ever be found for underrepresented fragments. In other words, *Ask Ernö* can’t learn to correctly predict spin systems that are not properly represented in the training set. The situation just described is reflected in the large lines of vertically aligned points, observed in Fig. 6. Most mistakes are located along these vertical series of points, proving that this was the main source of error in the test.

Other recurring mistakes can be related to underrepresented structures. For instance, the biggest errors for predictions based on 4-spheres (see Fig. 6, bottom) arose when the query returned a single matching fragment. In these cases the maximum uncertainty (20 ppm) given by the assigner to predictions based in less than 3 fragments allows for the propagation of an error that in principle should be rectified by new observations, but remains due to lack of the necessary data.

Detailed examples are given in the Additional file 4.

## Conclusions

The reduction in error and uncertainty and the increase in the amount of predictions proves that *Ask Ernö* is indeed improving its prediction capabilities as it iterates on the assignment-prediction cycle. After 10 iterations using a set of 2341 assignment problems, *Ask Ernö* was able to predict the chemical shifts of protons in a set of 298 molecules with an average error of no more than 0.265 ppm. At least 60 % of the chemical shifts were predicted with an error of less than 0.2 ppm. These are very promising results, especially for such a basic implementation of the concept.

It must be emphasized that Ask Ernö developed this capability fully autonomously: at no point it was fed with the fruits of the labour of human experts. The learning process of Ask Ernö is akin to that of a newcomer to the realm of NMR analysis, who is told the basic rules of assignment and through experience and induction develops his own NMR tables.

As expected, larger *n*-spheres provide better but fewer predictions. Furthermore, it was found that most errors occurred for underrepresented molecules when forcing highly uncertain predictions based on smaller fragments. For these reasons, it is expected that with more data the database could grow to a point where any query would match a large *n*-sphere. Thus, though the system currently tops at an average error of 0.265 ppm, the limit of <0.1 ppm error could be reached with enough data. Further improvements to this limit would require taking into account other experimental parameters such as solvent, concentration and temperature of acquisition, as major source of experimental errors.

Based on the results presented here, we expect to develop Ask Ernö into a state-of-the-art tool for automatic NMR analysis in the near future. Current efforts are focused in reforming the estimator of uncertainty in order to enhance the system’s capability to rectify its mistakes as it iterates. Correlation data from 2D experiments should also lead to significant improvement, when available.

## Availability of data and materials

The source code used in this work is available in the github repository https://github.com/cheminfo/autolearning. The dataset supporting the conclusions of this article is available in the github repository https://github.com/cheminfo/autolearning. The molecules in the dataset supporting the conclusions of this article are also included within the article (and its Additional file 2, Additional file 3) as sdf files.

## Additional files

10.1186/s13321-016-0134-6 Examples of spectra from the training set.
10.1186/s13321-016-0134-6 Molecules of the training set (format: molfile:.mol).
10.1186/s13321-016-0134-6 Molecules of the test set (format: molfile:.mol).
10.1186/s13321-016-0134-6 Example of errors occurring during the self-learning loop.

## Abbreviations

NMR: nuclear magnetic resonance
HOSE: hierarchically ordered spherical description of environment

## Footnotes

1 In reference to Ernö Pretsch and his classic book compiling thoroughly the necessary information for humans to assign spectra [21].
