# Supplementary material for: “Ask Ernö”: a self-learning tool for assignment and prediction of nuclear magnetic resonance spectra
Source: J Cheminform. 2016 May 5;8:26. doi: 10.1186/s13321-016-0134-6 (PMC4858875; doi:10.1186/s13321-016-0134-6)
Supplement: Supplementary file 1 — 10.1186/s13321-016-0134-6 Examples of spectra from the training set. [file 13321_2016_134_MOESM1_ESM.pdf]

Example 1

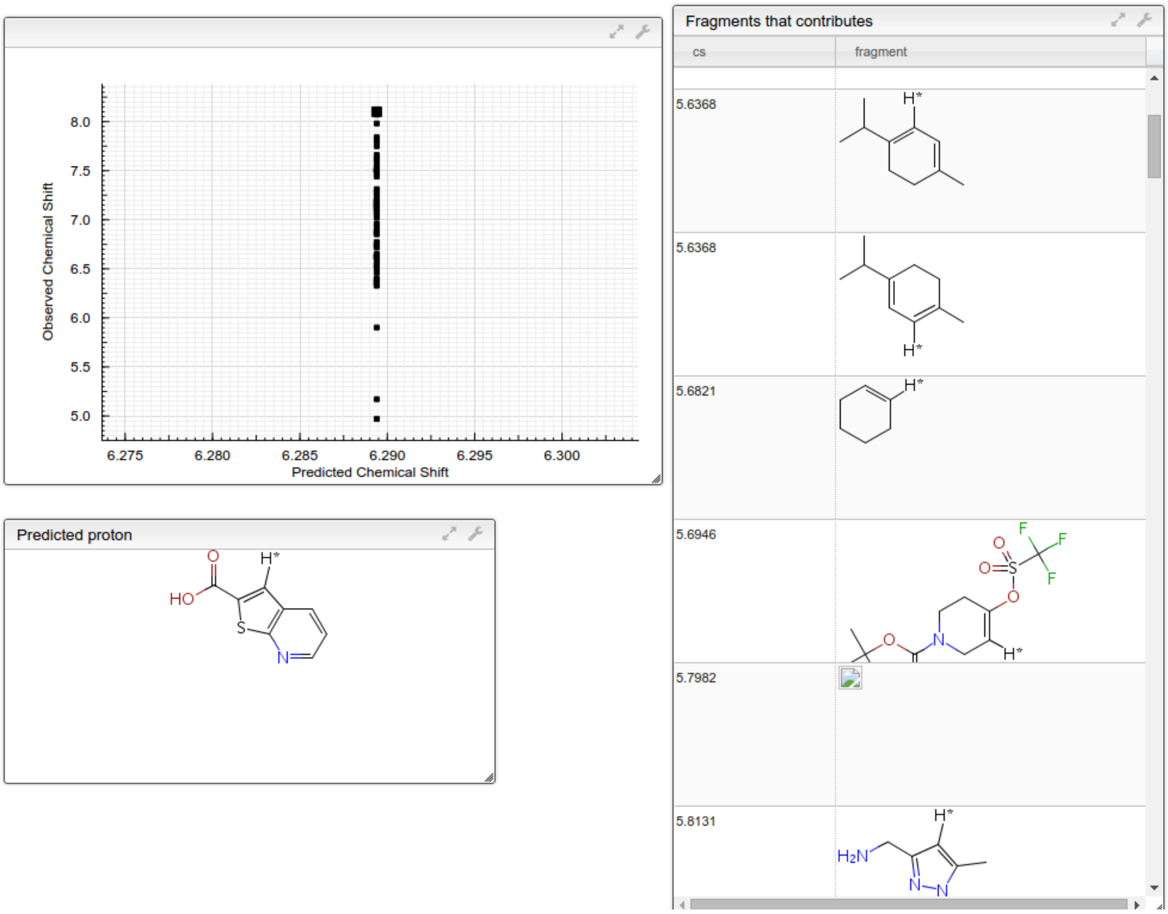

The image shows a predicted proton (left, bottom), its position in the predicted vs. observed chemical shift plane (left, top, larger square) and some of the molecules that contributed to its prediction. This chemical shift was predicted using a 2-sphere around the predicted proton (CH=CH-N), a fragment frequently found in organic compounds. As a consequence, the query for this fragment produced 73 hits, 6 of which are displayed in the picture. This causes the uncertainty of the prediction to be high (> 1 ppm), which unavoidably leads to errors.

The smaller squares arranged in a vertical line that are displayed in the upper-left plot correspond to the other predictions based on the same fragment. Note how their observed chemical shift values distribute around the predicted value. The highlighted error is then a random error unavoidable when small and frequently observed fragments are used for prediction.

Example 2

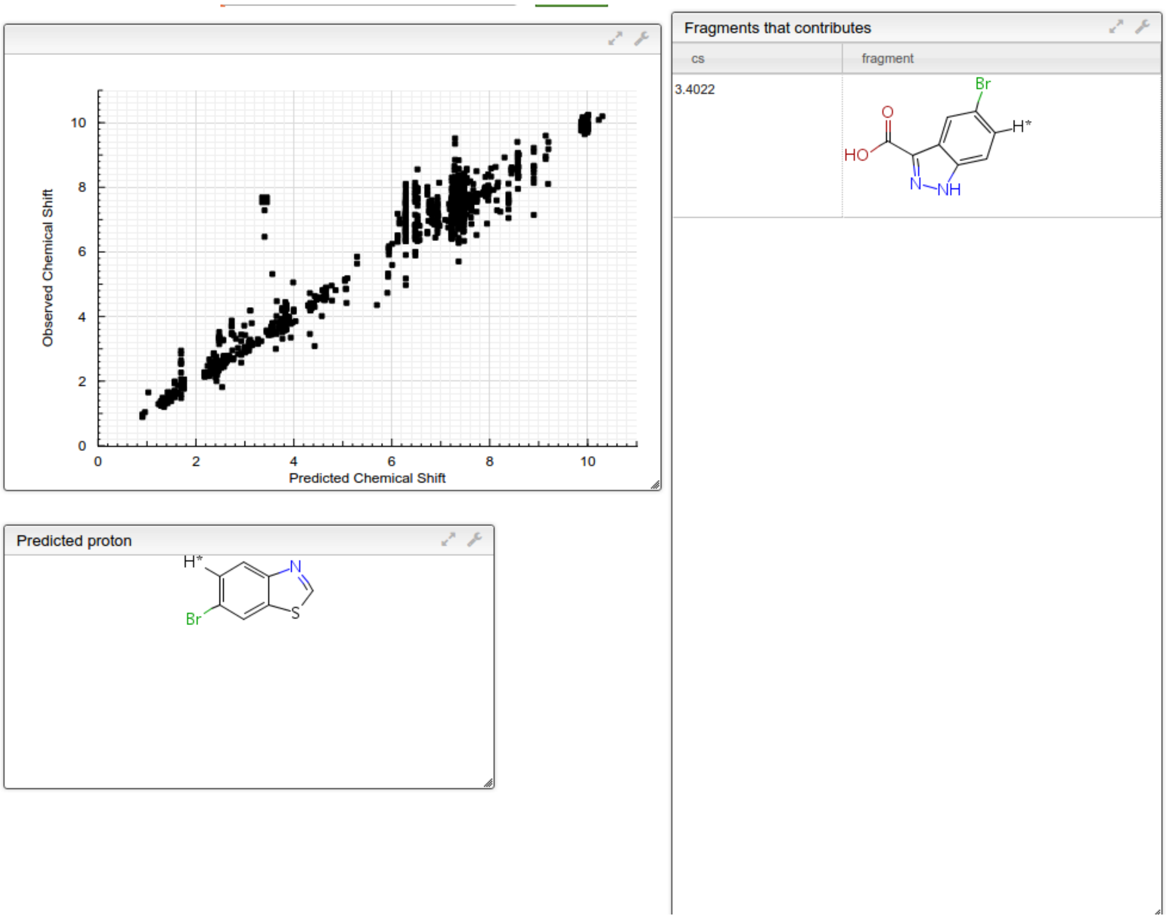

This time we are looking at a chemical shift prediction based on a single observation. The upper left plot shows the whole predicted vs. observed chemical shift plane, the case of study is highlighted as before (3.4 ppm, 7.6 ppm). Note that it is not aligned vertically with many other predictions; instead, it looks rather isolated. This is a sign that we are dealing with a different type of issue.

The lone database entry used for prediction (right) is wrong, an error that emerged by the mechanism illustrated in the previous example. This structure shares a 4-sphere with the proton whose chemical shift is being predicted (left, bottom) and it is the only database entry that does so. This causes *Ask Ernö* to take the prediction as completely uncertain (20 ppm), so any assignment reproducing the integration is acceptable for learning, regardless of the chemical shift error. By the end of this cycle the 4-sphere would be associated with two dissimilar chemical shifts and an average that is near neither observed value. In this way, the error introduced in the previous cycle propagates to the next. This error will remain until further training data is provided.
