# Supplementary figures and images for: “Ask Ernö”: a self-learning tool for assignment and prediction of nuclear magnetic resonance spectra
Source: J Cheminform. 2016 May 5;8:26. doi: 10.1186/s13321-016-0134-6 (PMC4858875; doi:10.1186/s13321-016-0134-6)

## Some molecules and spectra used for training

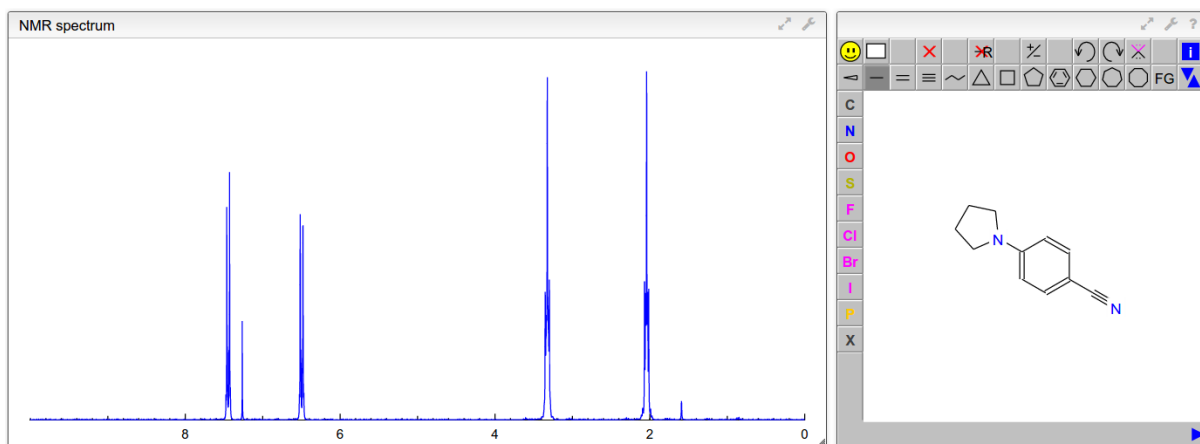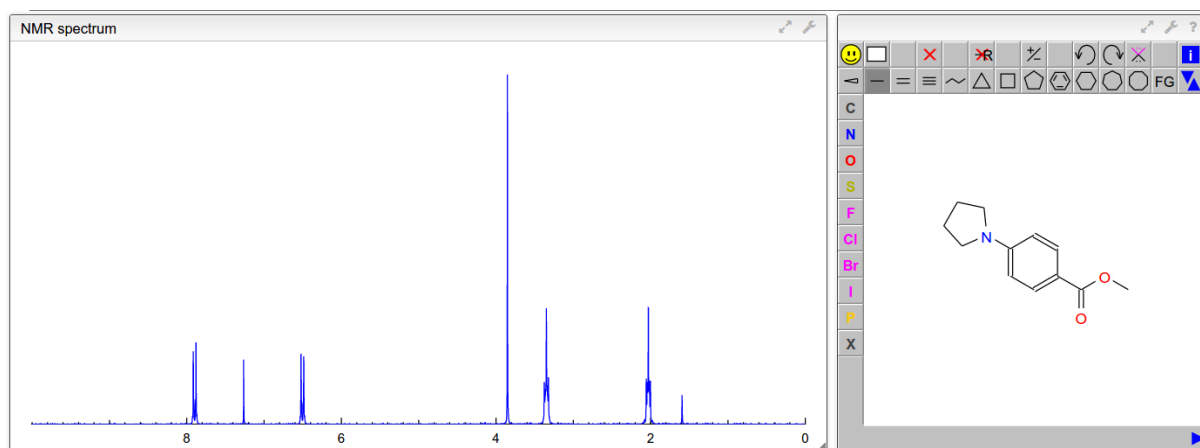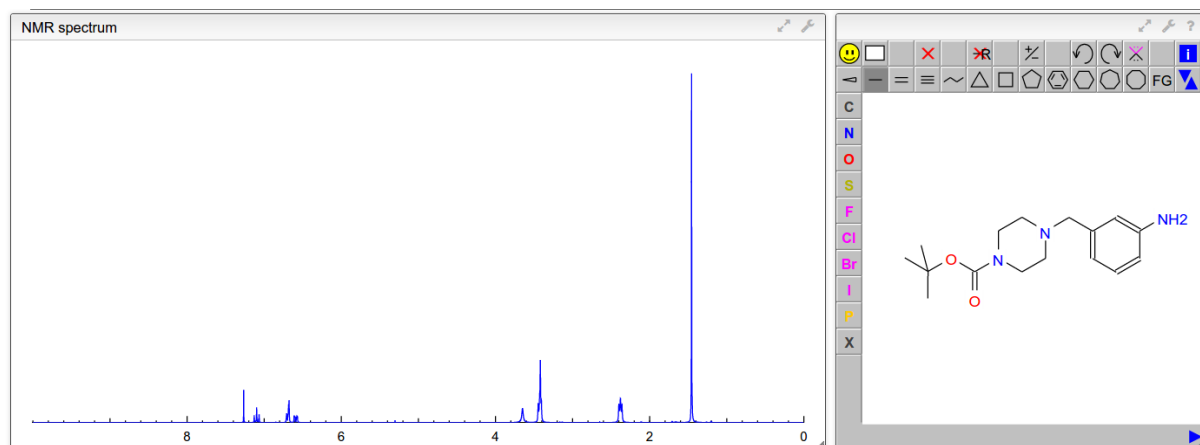

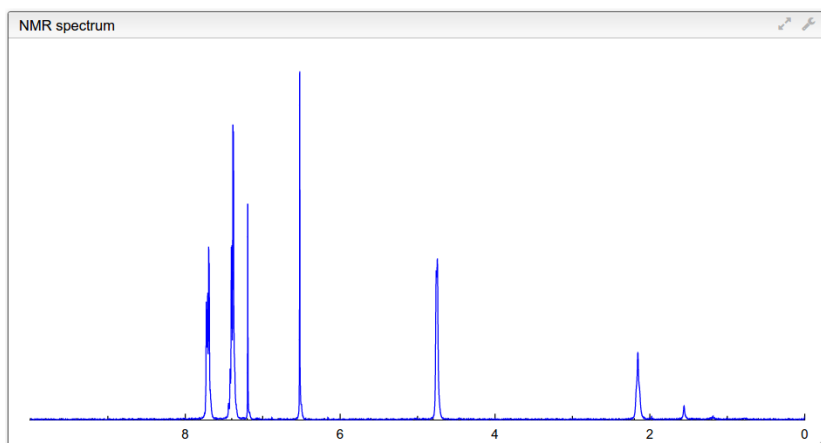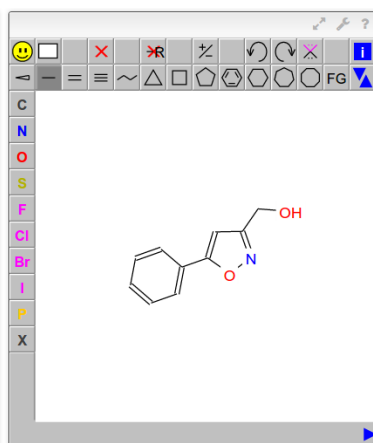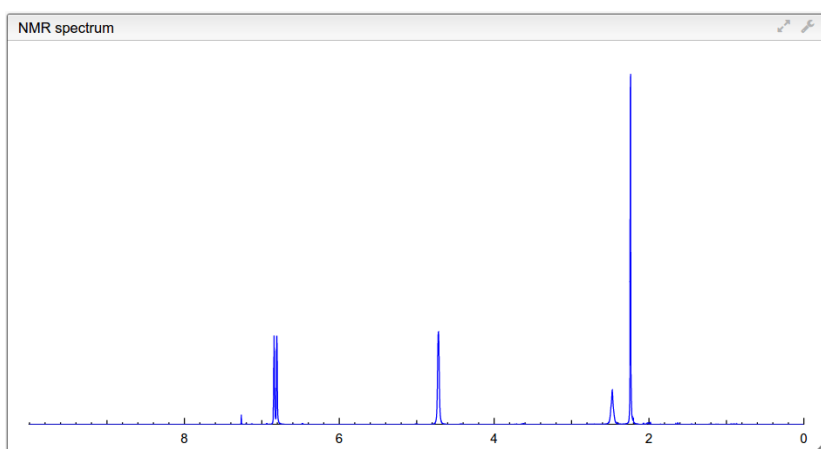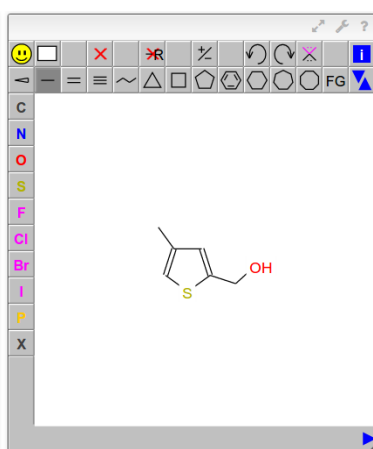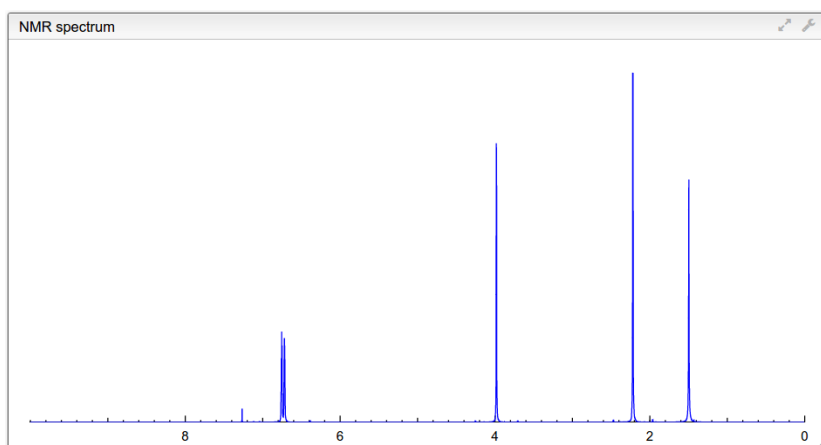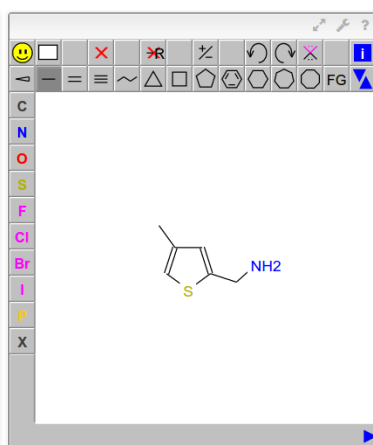

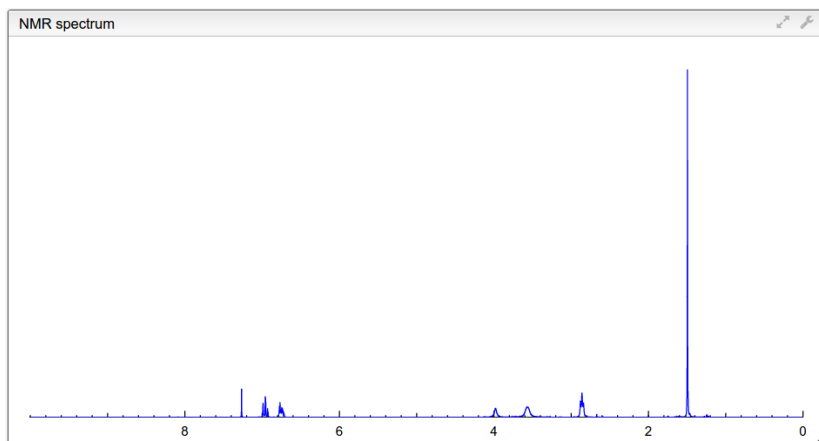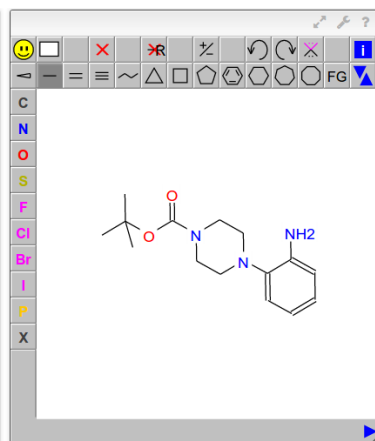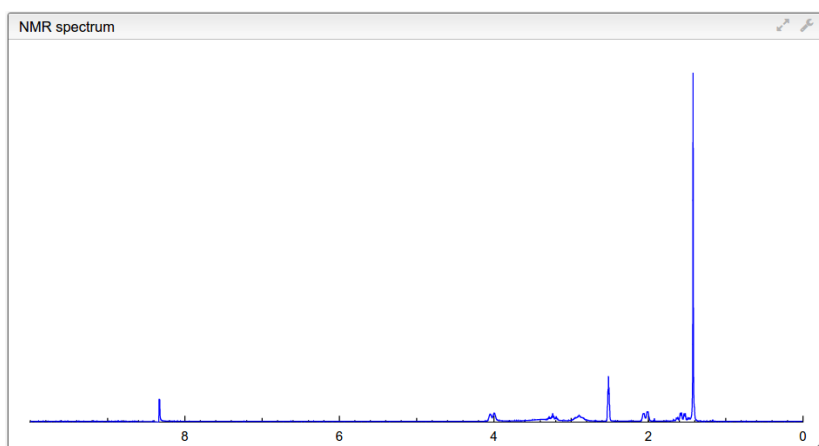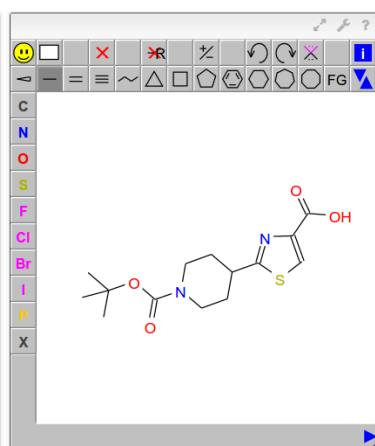

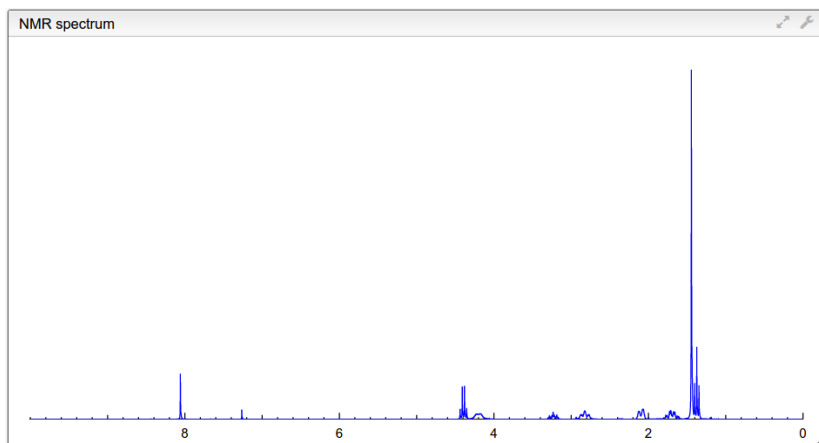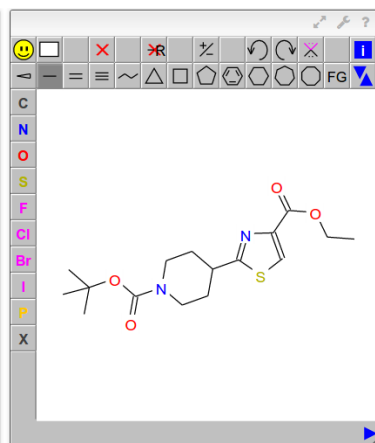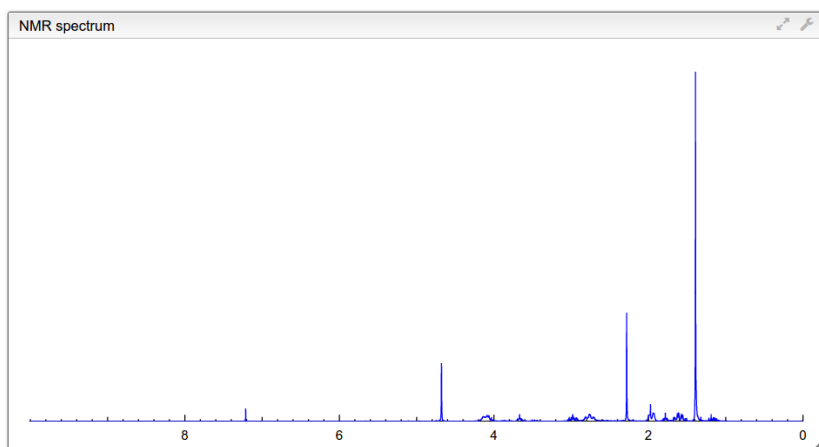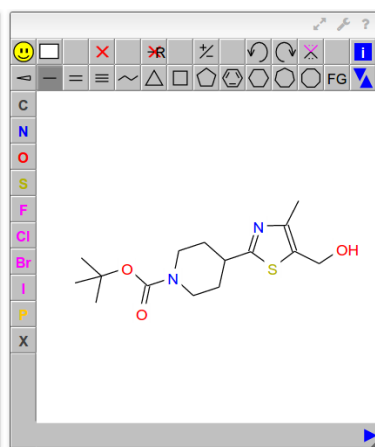

Supplement: Supplementary file 2 — 10.1186/s13321-016-0134-6 Molecules of the training set (format: molfile:.mol). [file 13321_2016_134_MOESM2_ESM.pdf]
